# Notes and Notices

**Published:** 1898-08

**Authors:** 


### NOTES AND NOTICES.

Dr. Givens' Sanitarium, at Stamford, Conn., for nervous and mental diseases, is one of the most favorably located in this country.

It is a quiet, homelike place, arranged on the cottage plan, where the rest cure, massage, faradization, galvanism, dieting, baths, and everything pertaining to the best treatment of patients requiring special care may be procured at reasonable prices.

Dr. Leverson's Note to Dr. Cory's Testimony, as published in the June number of The Homœopathic Physician, at pages 239 to 245, has been reprinted as a pamphlet, and can be supplied at ten dollars ($10.00) per thousand copies, or one dollar ($1.00) per hundred copies, postage extra.

Address The Homœopathic Physician,
1231 Locust St., Phila., Pa.

The International Hahnemannian Association, at its meeting in June last, elected the following officers for the year 1898-99: President, Walter M. James, Philadelphia, Pa.; Vice-President, C. M. Boger, Parkersburg, W. Va.; Secretary, Erastus E. Case, Hartford, Conn.; Treasurer, Franklin Powel, Chester, Pa.; Corresponding Secretary, Lawrence M. Stanton, New York City; Censors—B. L. B. Baylies, Brooklyn, N. Y.; B. Fincke, Brooklyn, N. Y.; C. Carleton Smith, Philadelphia, Pa.; Annie L. Geddes, Montclair, N. J.; Alice B. Campbell, Brooklyn, N. Y.; Bureau Chairmen—Materia Medica, C. M. Boger, Parkersburg, W. Va.; Clinical Medicine, W. A. Yingling, Emporia, Kan.; Obstetrics, J. A. Tomhagen, Chicago, Ill.; Surgery, T. S. Hoyne, Chicago, Ill.; Necrologist, John Storer, Chicago, Ill.

Longport, New Jersey, published by A. H. Phillips & Co., Real Estate dealers, 1315 Atlantic Avenue, Atlantic City, New Jersey. Price ten cents.

This little pamphlet of 16 pages is issued to set forth the advantages of Longport a small town on the southern end of the island called Absecon Beach; its northern end being occupied by Atlantic City. It has the Atlantic Ocean on one side and Great Egg Harbor Bay on the other side. The pamphlet is embellished with beautiful photogravures of the ocean, the landscape, and the bay.

The Editorial this month was left out to make room for the very important articles which are now published. The article copied from our English contemporary, *The Monthly Homœopathic Review*, upon the opening of Hahnemann's tomb is especially interesting, and we felt justified in copying it entire.—[Ed.]

